# Delayed β-cell response and glucose intolerance in young women with Turner syndrome

**DOI:** 10.1186/1472-6823-11-6

**Published:** 2011-03-15

**Authors:** Britta E Hjerrild, Jens J Holst, Claus B Juhl, Jens S Christiansen, Ole Schmitz, Claus H Gravholt

**Affiliations:** 1 Department of Endocrinology and Internal Medicine and Medical Research Laboratories, Aarhus Sygehus NBG, Aarhus University Hospital, Denmark; 2 Department of Biomedical Sciences, The Panum Institute, University of Copenhagen, Denmark; 3 Department of Endocrinology and Diabetes, Odense University Hospital, Denmark

## Abstract

**Background:**

To investigate glucose homeostasis in detail in Turner syndrome (TS), where impaired glucose tolerance (IGT) and type 2 diabetes are frequent.

**Methods:**

Cross sectional study of women with Turner syndrome (TS)(n = 13) and age and body mass index matched controls (C) (n = 13), evaluated by glucose tolerance (oral and intravenous glucose tolerance test (OGTT and IVGTT)), insulin sensitivity (hyperinsulinemic, euglycemic clamp), beta-cell function (hyperglycaemic clamp, arginine and GLP-1 stimulation) and insulin pulsatility.

**Results:**

Fasting glucose and insulin levels were similar. Higher glucose responses was seen in TS during OGTT and IVGTT, persisting after correction for body weight or muscle mass, while insulin responses were similar in TS and C, despite the higher glucose level in TS, leading to an insufficient increase in insulin response during dynamic testing. Insulin sensitivity was comparable in the two groups (TS vs. control: 8.6 ± 1.8 vs. 8.9 ± 1.8 mg/kg*30 min; p = 0.6), and the insulin responses to dynamic β-cell function tests were similar. Insulin secretion patterns examined by deconvolution analysis, approximate entropy, spectral analysis and autocorrelation analysis were similar. In addition we found low IGF-I, higher levels of cortisol and norepinephrine and an increased waist-hip ratio in TS.

**Conclusions:**

Young normal weight TS women show significant glucose intolerance in spite of normal insulin secretion during hyperglycaemic clamping and normal insulin sensitivity. We recommend regularly testing for diabetes in TS.

**Trial Registration:**

Registered with http://clinicaltrials.com, ID nr: NCT00419107

## Background

Turner syndrome (TS) is usually associated with reduced adult height and gonadal dysgenesis, premature ovarian failure and infertility. Increased morbidity has been reported with an increased risk of congenital and acquired cardiovascular disease, thyroid disease, osteoporosis and diabetes. Early reports of impaired glucose tolerance (IGT) in TS [1,2] have been followed by studies finding several abnormalities of the glucose metabolism in both girls [3] and women [4,5] with TS. Epidemiological studies have shown an increased risk of developing both type 1 diabetes (relative risk: 11.6) and type 2 diabetes (T2DM)(relative risk: 4.4) [6], in addition to increased mortality due to diabetes [7,8].

IGT is present in 25-78% of adult TS populations evaluated by oral glucose tolerance testing (OGTT) [4,5], and seems to be more prevalent in TS compared to both healthy controls and women with premature ovarian failure and thus reduced oestrogen exposure [5]. Other studies have suggested the presence of reduced insulin sensitivity [3,9] or impaired beta-cell function [4,5]. However, the exact mechanism behind the increased occurrence of type 2 diabetes is not clear.

Our aim was to establish the separate roles of insulin sensitivity and β-cell function on glucose homeostasis in young women with TS compared to BMI and age matched controls. We hypothesized that early β-cell failure would be present and possibly aggravated by insulin resistance.

## Methods

The study group consisted of 13 women with TS verified by karyotyping and 13 age- and BMI-matched control women. All TS but one had the karyotype 45, X, one had 45, X/46, X, del(X). Seven of the participants had earlier received growth hormone therapy. All participants but one in the TS group completed all study days.

The patients were recruited consecutively through the National Society of Turner Contact Groups in Denmark. All patients received hormone replacement therapy (HRT).

Exclusion criteria were known diabetes, BMI above 30, untreated hypo- or hyper-thyroidism, present or past malignant disease, symptomatic heart disease or daily use of prescribed medicine known to affect glucose metabolism other than HRT. Control women did not use any prescribed medicine including hormonal contraception.

All participants received oral and written information concerning the study prior to giving their written informed consent. The protocol was carried out in accordance with the Helsinki declaration and approved by the Aarhus County Ethical Scientific Committee (no. 20040108).

**Figure 1:**
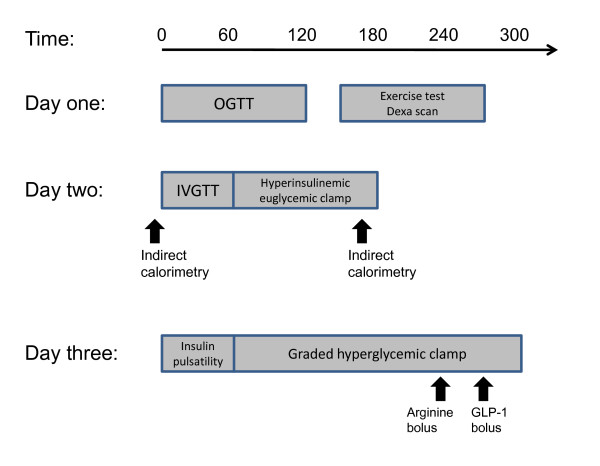
**Study design of day 1, 2 and 3**. On day 1 an oral glucose tolerance test (OGTT), as well as DEXA scan and exercise test was performed. On day 2, we did the intravenous glucose tolerance test, followed by a hyperinsulinemic euglycemic clamp. Energy expenditure was assessed in fasting condition and during the steady state period of the clamp. On day 3, we performed insulin pulsatility testing followed by a graded hyperglycaemic clamp with arginine bolus and finally GLP-1 bolus to stress the β-cell maximally (for details - see Materials and Method section).

Participants were examined over three days, day one and two being consecutive days. The final examination day was performed more than four weeks after day two. Participants met in the morning after an overnight fast from 10 pm the previous night on all three days, without engaging in major physical exercise for 48 hours before the investigations (Figure 1). The women were examined independent of the period of their menstrual cycle.

### Body Composition

On day one, body weight, height, waist-to-hip ratio and blood pressure was measured. Total and regional fat mass (FM) (g), and lean body mass (LBM) (g) was measured by dual-energy X-ray absorptiometry (DXA) using a Hologic QDR scanner (Hologic, Inc., Waltham Mass, version 7.20D). Cross calibration was ensured through the use of double measurements and a common phantom [10].

### OGTT and Vo_2max_

An OGTT was performed, administering 75 g glucose orally. Insulin, C-peptide, free fatty acids (FFA), triglycerides, growth hormone (GH), IGF-I, glucagon-like peptide 1 (GLP-1) and glucose-dependent insulinotropic polypeptide (GIP) were measured at baseline and at 15, 30, 45, 60, 90, 120 min. Serum insulin and plasma glucose responses were measured as area under the curve (AUC) and as the incremental AUC (iAUC) using the trapezoidal rule.

A 6 min sub-maximal exercise test on a bicycle ergometer (Monark Ergomertric 829 E, Monark, Varberg, Sweden) was performed for estimation of Vo_2max _.

### IVGTT and Indirect Calorimetry

On day two intravenous catheters (Venflon, Viggo AB, Helsingborg; Sweden) was placed in an antecubal vein for infusion and in an arterialized hand vein for blood sampling. After 30 minutes bed-rest, baseline indirect calorimetry was performed measuring resting energy expenditure (EE) and respiratory quotient during 20 min (Deltatrac monitor, Datex Instrumentarium, Helsinki, Finland) [11]. An IVGTT was performed and glucose (25 g as 50% glucose) was administered as a bolus dose within 90 sec and subsequent blood samples were drawn at 0, 4, 8, 10, 12, 15, 18, 20, 25, 30, 40, 50 and 60 min.

### Hyperinsulinemic Euglycemic Clamp

The IVGTT was followed by a hyperinsulinemic euglycemic clamp as previously described [12]. At 60 min infusion of 1.0 mU/kg/min insulin was initiated and infusion of 20% glucose was adjusted to stabilize plasma glucose around 5.0 mmol/l. Minutes 150-180 were considered as a hyperinsulinemic steady state and during this period the indirect calorimetric measurement was repeated and samples of blood were drawn every 15 min.

### Insulin Pulsatility

Initially on study day three spontaneous insulin secretion was recorded by measuring insulin levels every minute for 60 minutes. Serum insulin concentration time series were evaluated by deconvolution analysis, autocorrelation analysis, spectral analysis and approximate entropy.

#### Deconvolution analysis

Serum insulin concentration time series were analyzed in a blinded manner by deconvolution analysis to quantitate insulin secretory burst mass, burst amplitude, basal secretion, and interpulse interval [13,14].

#### Detrending

To eliminate the effects of nonstationarity in the data, approximate entropy (ApEn), spectral analysis, and autocorrelation analysis were performed on the residuals, after subtraction of an 11-point centered moving average process [15]. This length of the moving average process was chosen to ensure optimal detrending.

#### ApEn

ApEn measures the likelihood that patterns repeat throughout the time series [15]. By application of a small r value (*e.g*. r = 0.2 sd), ApEn evaluates fine (sub) patterns in the time series, and a larger r value (*e.g*. r = 1.0 sd) is applied to evaluate more coarse patterns. A higher ApEn value indicates a more irregular time series.

#### Spectral analysis and autocorrelation analysis

By spectral analysis insulin concentration time series is described by sinus waves of different frequencies. The predominant frequency and the density hereof was recorded. A Tukey window of 25 data points was used, and spectra were normalized, assuming that the total variance in each time series was 100%, enabling comparison of spectral estimates despite the different absolute insulin values. Autocorrelation analysis was performed and the lag time and the maximal autocorrelation coefficient were recorded. All data analyses were performed in a blinded manner.

### Graded Hyperglycemic Clamp, Arginine and GLP-1 bolus

Following insulin pulsatility testing, a graded hyperglycemic clamp was applied on day three. At 60 minutes glucose levels were raised to 7 mmol/l by the use of between 2 and 5 ml 50% glucose and continuous infusion of 20% glucose, to raise glucose as quickly as possible and clamp glucose at levels 7, 9, 10, 11 and 12 mmol/l for 30-minute intervals. After 30 min at glucose level of 12 mmol/l a 5 g i.v.-bolus of arginine was administered measuring the effect on baseline parameters every 5 minutes for 30 minutes. This was followed by an iv-bolus of 2.5 nmol GLP-1 and the effect was likewise monitored for 30 min.

### Assays

Plasma glucose was measured in duplicate immediately after sampling on a Beckman Glucoanalyzer (Beckman Instruments, Palo Alto, CA, USA). Serum insulin was measured by ELISA employing a two-site immunoassay (DakoCytomation, Cambridgeshire, United Kingdom), C-peptide by ELISA (DakoCytomation, Cambridgeshire, United Kingdom), serum FFA by a colorimetric method (Wako Pure Chemical Industries, Neuss, Germany), plasma TG by COBAS Fara II, serum GH and cortisol by time-resolved flouroimmunoassay (AutoDELFIA, PerkinElmer, Wallac, Turku, Finland). Total IGF-I was analysed as previously described [16]. Epinephrine and norepinephrine were measured by HPLC [17], glucagon by an in-house radioimmunoassay, GLP-1 by a C-terminal radioimmunoassay [18] and total GIP as previously described [19].

### Statistical methods

The number of participants was determined from a power calculation based on a minimal difference in glucose levels (area under the curve (AUC)) during an OGTT of 4.4 mmol/L/2 hours in women with TS and controls and estimating an α of 0.05 and a power of 80% [4], which lead to a minimum sample size of 12. Using data on insulin sensitivity in TS estimated by a euglycemic hyperinsulinemic clamp [9], would have yielded a minimal sample size of 8.3. All statistical calculations were done using SPSS 15.0. Insulin and glucose response to varying stimuli were compared by area under the curve (AUC) computation. Results are expressed as mean ± standard deviation (SD). For inter group comparison a paired test was applied since TS and controls were closely matched individually on age and BMI. P-values less than 5% were considered significant.

## Results

### Anthropometry and energy expenditure

The close matching of TS and controls resulted in comparable age, BMI, FM (%) and LBM (%) (Table 1). Waist-hip ratio and maximal oxygen uptake were higher in TS (Table 1).

**Table 1:** Anthopometric and clinical data on study participants.

	TS (N = 13)	Control (N = 13)	
Age (years)	33.2 ± 4.8	33.7 ± 5.5	NS
Height (cm)	150.1 ± 6.8	168.3 ± 6	< 0.01
Weight (kg)	55.5 ± 8	69.6 ± 10.5	< 0.01
BMI (kg/m^2^)	24.6 ± 3	24.5 ± 3.1	NS
Waist (cm)	78.3 ± 7.7	78.1 ± 8.4	NS
Hip (cm)	94.5 ± 7	105.9 ± 7.8	<0.01
Waist-hip ratio	0.83 ± 0.06	0.74 ± 0.06	<0.01
Systolic blood pressure (mmHg)	115.1 ± 11.4	116.5 ± 9.2	NS
Diastolic blood pressure (mmHg)	73.6 ± 12.4	70.4 ± 9.2	NS
Vo2max (mlO_2_/kg)	45.2 ± 7.2	37.6 ± 8.2	<0.004
FM (kg)	17.7 ± 4.6	21.5 ± 6.6	0.02
FM (%)	32.3 ± 5.8	31.2 ± 5.6	NS
LBM (kg)	34.6 ± 4.5	44 ± 5.2	<0.01
LBM (%)	64.4 ± 5.4	65.3 ± 5.2	NS

Antroprometric data, blood pressure and bocycomposition data from TS women and controls. Values are mean ± SD. Fat mass (FM) and lean body mass (LBM) are shown as total (kg) and percentage (%).

Energy expenditure (EE) was similar in TS and controls in both basal state and during the clamp steady state (data not shown). No difference was found in EE change (ΔEE) (ΔEE 103.8 ± 74.2 vs. 123.1 ± 46.3; p = 0.5). No significant difference in respiratory quotient (RQ) was present between groups, in neither basal nor steady state conditions (basal: p = 0.3; steady state: p = 0.4).

### OGTT

**Figure 2:**
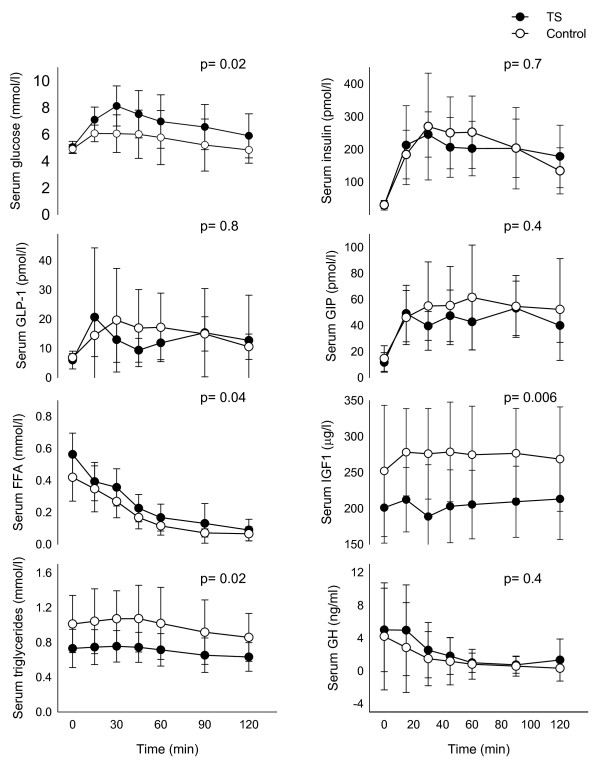
**Levels of glucose, insulin, glucagon-like**. peptide-1 (GLP-1), glucose-dependent insulinotropic polypeptide (GIP); free fatty acids (FFA), triglycerides, Insulin-like growth factor 1(IGF-1) and growth hormone (GH) during the 2-hour oral glucose tolerance test. P-values are based on differences between TS and controls based on AUC and given in the figure. All mean ± SD.

Fasting levels and 2 hour levels of glucose and insulin were similar in TS and controls (fasting glucose: p = 0.4; 2 h glucose: p = 0.06; fasting insulin: p = 0.3; 2 h insulin: p = 0.2), while AUC_glucose _was higher in women with TS, but AUC_insulin _was comparable, indicating a relatively lower insulin response in TS (Figure 2) and correcting for total bodyweight or LBM did not change this (results not shown). IGT (2 h glucose > 7.6 mmol/l) was present in two women with TS and no controls. The ratio AUC_insulin _/AUC_glucose _was almost significantly different between groups (TS vs. control: 28.7 ± 6.1 vs. 36.8 ± 10.8; p = 0.054). Results from analysis of C-peptide data were similar to insulin results (p = 0.08). Exclusion of the 2 TS subjects with IGT and their controls did not change these findings.

Triglycerides levels were significantly lower among TS (AUC_trigly_: 85.4 ± 22.1 vs. 118.7 ± 42.5 mmol/L*2 h; p = 0.004) (Figure 2). Likewise IGF-I was lower in TS (AUC_IGF-1_: 24970 ± 5792 vs. 33023 ± 7619 μg/L*2 h; p = 0.004), a difference already present at baseline (IGF-1_t = 0_: 201 ± 49 vs. 252 ± 91 μg/L; p < 0.001). No significant difference was found in FFA, GLP-1, GIP or GH levels during the OGTT (Figure 2).

### IVGTT

The 60 minutes glucose response to intravenous glucose (25 g) was higher in TS vs. controls (p < 0.001), even after correction for bodyweight (BW) (AUC_glucose corBW_: 11.8 ± 2.4 vs. 7.7 ± 1.5 mmol/l*60 min*kg; p < 0.001), suggesting a reduced ability to respond to a glucose load. Both first phase insulin response (AUC_0-10 min_, 2940 ± 1355 vs. 2703 ± 922 pmol/l*10 min, p = 0.6) and the 60 minutes insulin response were similar in the groups (AUC_0-10 min_, 10289 ± 4589 vs. 8343 ± 1950 pmol/l*60 min, p = 0.2), despite higher glucose levels among TS. When corrected for bodyweight there was, however, a higher insulin response (0-60 min) in TS compared to controls (AUC_insulincorBW_: 190 ± 90 vs. 121 ± 27 pmol/l*60 min*kg; p = 0.03), as would be expected due to the higher glucose levels in TS. This finding did not persist if the two women with TS and IGT and their corresponding controls were excluded. The weight adjusted first phase insulin response (0-10 min) was, however, still similar in the two groups (AUC_insulincorBW_: 53 ± 24 vs. 39 ± 12 pmol/l*10 min*kg; p = 0.07). Calculation of a glucose/insulin ratio revealed no difference between groups (ratio AUC_insulin_/AUC_glucose_: 16.5 ± 8.1; 16.0 ± 3.6; p = 0.9). No difference was found in FFA levels at baseline or during the IVGTT (baseline: p = 0.5; AUC_FFA_: p = 0.4).

### Hyperinsulinemic euglycemic clamp

The insulin stimulated glucose uptake (M-value) obtained from the hyperinsulinemic, euglycemic clamp (8.6 ± 1.8 vs. 8.9 ± 1.8 mg/kg*30 min; p = 0.6) and uptake per LBM (13.7 ± 2.7 vs. 14.0 ± 2.2 mg/kg LBM*30 min; p = 0.7) were similar in the two study groups. The insulin levels during the last 30 minutes of the clamp, were slightly higher in controls (338 ± 56 vs. 380 ± 45 nmol/l; p = 0.04). The disposition index calculated using iAUC and the M-value was not significantly different between groups (p = 0.4).

Cortisol levels were elevated in TS both at baseline (365 ± 162 vs. 243 ± 112 nmol/l; p = 0.04) and during the steady state of the hyperinsulinemic clamp (AUC_cortisol_: 7828 ± 3699 vs. 5128 ± 2046 nmol/l*30 min; p = 0.03), as was steady state norepinephrine (AUC_norepinephrine_: 7421 ± 1769 vs. 6037 ± 1802 pg/ml*30 min; p = 0.04). Levels of glucagon (AUC_Glucagon_: 599 ± 519 vs. 584 ± 179 pg/ml*30 min, p = 0.9), GH (AUC_GH_: 26 ± 38 vs. 69 ± 113 ng/ml*30 min, p = 0.2) and epinephrine (AUC_epi_: 1533 ± 2054 vs. 1269 ± 457 pg/ml*30 min, p = 0.7) were similar during the steady state period of the clamp.

### Insulin Pulsatility

The overall insulin release was comparable between TS and control women, with similar burst mass and amplitude, as well as basal secretion. The regularity of the insulin release pattern, as assessed by approximate entropy was similar, as was the spectral power and the autocorrelation coefficient (Table 2).

**Table 2:** Insulin pulsatility analysis.

	TS (N = 12)		Control (N = 13)		
	
	Mean ± SD	Range	Mean ± SD	Range	P-value
Deconvolution					
Mass (pmol/L/pulse)	12.14 ± 3.93	7 - 17.5	15.49 ± 8.1	8 - 38.2	0.2
Amplitude (pmol/L*min)	9.67 ± 3.15	5.5 - 13.9	12.35 ± 6.46	6.4 - 30.5	0.2
Pulse interval (min/pulse)	7.92 ± 1.71	6.1 - 12.4	7.49 ± 0.95	6 - 9.5	0.3
Basal secretion (pmol/L*min)	4.63 ± 2.25	4.48 - 2.09	4.48 ± 2.09	1.7 - 7.3	0.7
Pulse fraction	0.28 ± 0.11		0.33 ± 0.11		0.1
Regularity Analysis					
ApEn (m = 1, r = 0.2 × SD)	1.34 ± 0.15	0.974 - 1.503	1.39 ± 0.058	1.32 - 1.51	0.3
ApEn (m = 1, r = 1.0 × SD)	0.586 ± 0.19	0.035 - 0.772	0.637 ± 0.073	0.5 - 0.762	0.4
Spectral power	7.03 ± 2.85	3.3 - 11.9	7.58 ± 2.2	4.3 - 12.8	0.4
Spectral frequency	10.58 ± 2.27	8 - 13	10.69 ± 2.39	7 - 13	0.6
Autocorrelation coefficient	0.198 ± 0.144	0.005 - 0.462	0.246 ± 0.129	0.05 - 0.468	0.3
Autocorrelation frequency	9.0 ± 2.8	6 - 15	9.62 ± 2.29	6 - 13	0.05

Parameters of pulsatility analysis based on deconvolution analysis, spectral analysis, and autocorrelation analysis and on the estimation of approximate entropy (ApEn) of individual insulin concentration time series in TS and controls.

### Graded hyperglycaemic clamp, arginine and GLP-1 bolus

**Figure 3:**
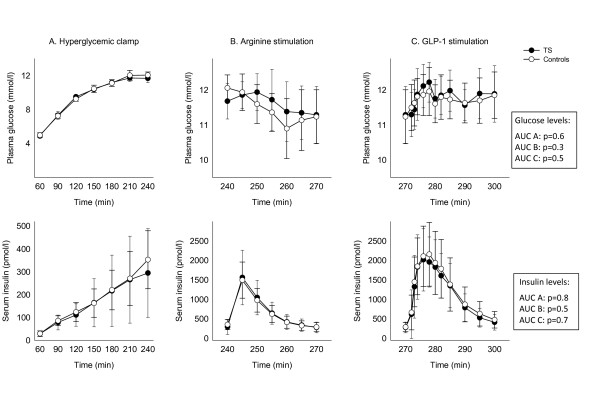
**Glucose and insulin levels (mean ± SD) during the hyperglycemic clamp (60-240 minutes) (A), arginine (240-270 minutes) (B) and GLP-1 stimulation (270-300 minutes) (C)**. P-values are given in the figure.

**Figure 4:**
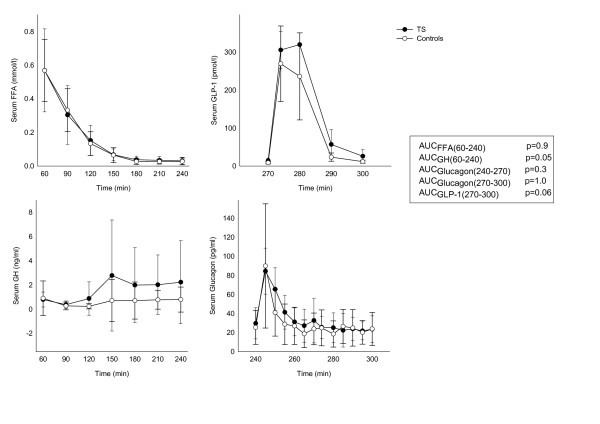
**Growth hormone (GH) and free fatty acid (FFA) during the hyperinsulinemic clamp period (60-240 minutes)**. Glucagon levels during the arginine and GLP-1 stimulation (240-300 minutes), and GLP-1 levels during the GLP-1 infusion period (270-300 minutes). All numbers are presented as mean ± SD. P-values are given in the figure.

Beta-cell function was measured as the insulin response to hyperglycaemia, arginine and GLP-1 stimulation. During the entire stimulation period the levels of glucose and insulin were identical in the groups (Figure 3). No significant difference in FFA or GH levels was present during the hyperglycaemic clamp, and glucagon levels during arginine and GLP-1 stimulation were similar between groups (Figure 4). GLP-1 was measured to verify a sufficient response to the bolus injection.

## Discussion

The salient results of the present study are the discrete perturbations in the glucose handling in young females with Turner syndrome. We found an increased level of glucose during 1) the OGTT and 2) during the IVGTT, and an insufficient concomitant compensatory increase in the level of insulin, resulting in a reduced insulin-to-glucose ratio. The study provides a detailed assessment of the glucose homeostasis in TS, and it extends and refines the results of previous studies. Study participants in the present study were matched closely, not only on age and BMI, but also on body fat. Using the gold standard for determining insulin sensitivity, the euglycemic clamp, we found no difference between TS and controls.

The backdrop for the present study is epidemiological data showing a four-fold increase in the risk of T2DM [6]. Similar fasting glucose and insulin levels are present in TS and controls [4,20,5,21]. Previous studies of insulin sensitivity in TS have shown equivocal results, in part due to an insufficient match on body composition. Reduced insulin sensitivity among children [3,22] and adults [9] has been reported, but here TS had significantly higher BMI compared to controls. Studies of BMI and fat mass matched groups of TS and controls have not found insulin resistance to be an important trait in TS [4,5,21]. Deficits in glucose metabolism seem to be present after glucose stimulation, and it has repeatedly been demonstrated that the response to oral glucose stimulation is impaired in TS [4,5,23]. In the present study 2 (18%) participants with TS had IGT, none in the control group. Impaired glucose tolerance was not an exclusion criterion in this study, which can be disputed, but this would exclude up to 50% of the TS population, resulting in a group of TS which would not be representative for the population. Leaving the two TS with IGT out of the data analysis, however, only changed the results marginally in regard to the OGTT, but not in regard to the results from the IVGTT.

To our knowledge β-cell function in this patient group has not been studied thoroughly before. The β-cell function in theory depends on: 1) the total β-cell mass, 2) the sensitivity of the individual cells to the stimulus and 3) the secretory capacity of the individual cell [24]. Intravenously administered hyperglycaemia, followed by first arginine, then GLP-1, resulted in an insulin response of the same magnitude in TS and controls. However, the first phase insulin response (0-10 min) during the IVGTT demonstrates a reduced insulin peak relative to the glucose peak in TS. This could suggest that the discrete reduction in insulin secretion following a glucose load is time dependant and becomes apparent after an acute glucose load, due to a delay in insulin secretion. This has earlier been described in a small TS population tested by OGTT [25], as also shown here with an increased AUC of glucose but comparable AUC for insulin compared with controls during the OGTT. By closely matching on age and BMI we have eliminated some of the confounding factors of IGT not directly linked to TS. Recently, Bakalov et al found a 25% prevalence of T2DM among a large group of adult TS tested by OGTT (n = 224) with a mean age of 35 years [23], corroborating epidemiological evidence [6-8]. They went on to divide their study group by karyotype into patients with delXq with a T2DM rate of 9% (similar to the T2DM rate in the background population), 45, X with a rate of 18%, delXp with a rate of 23%, and impressively, a rate of 43% among subjects with isochromosome Xq. These data suggests that haploinsufficiency of genes on Xp increases the risk of T2DM to 18-23%, and additionally that haploinsufficiency of Xp combined with trisomy for Xq genes (karyotypes with isochromosome Xq) further increases the risk of T2DM [23]. Gene expression profiling of 45, X and isochromosome Xq groups suggested overexpression of transcription factors involved in diabetes, pancreatic islet and β-cell function, as well as proinflammatory action in patients with isochromosome Xq and mirrored by increased levels of circulating CRP, IGF2 and GAD antibodies. Increased CRP [26,21]and GAD antibodies has been found before and the latter has been linked to the presence of isochromosome Xq[27]. Likewise, it is well known that the rate of autoimmunity in general is hugely increased in TS [28] being most pronounced for females with isochromosome Xq [28], and possibly also affected by the presence of allelic variation of the other genes on other chromosomes, such as PTPN22 gene [29].

During the IVGTT a standard dose of glucose was given, which means that participants with TS and a smaller body size received a relatively higher dose of glucose compared to controls. However, one would have expected a similar glucose level, with an appropriately increased level of insulin among TS. The present and previous results point towards a relative inability of patients with TS to respond appropriately to a glucose load, however not dependent on the prevailing estradiol level, since the defect in glucose handling is present both during basal circumstances and during hormone substitution therapy [4].

During the OGTT triglycerides were lower among TS, as was serum IGF-I, as also seen in previous studies where we found evidence of perturbation of the entire GH-IGF-IGFBP axis [30-32]. Interestingly, a recent study in children with TS showed that previously GH treated girls had less subcutaneous and visceral fat and less glucose intolerance than GH naïve girls, pointing towards either a long lasting protective effect of GH treatment on body composition and glucose homeostasis or that GH treatment actually corrected some underlying GH deficiency or a combination of both [33].

In addition we found higher levels of cortisol and norepinephrine during the euglycemic clamp. The elevation of cortisol may well be due to HRT induced elevation of cortisol binding globulin, which leads to elevated total cortisol (measured here), but does not increase free cortisol [34]. Elevated norepinephrine leves has been described before and linked to dysregulation of sympathetic nervous system and resting tachycardia [35]. Taken together, these data from the OGTT, IVGTT and clamp studies, suggests that a number of variables, such as prevailing levels of IGF-I (and indeed the entire GH-IGF-IGFBP axis [32]), norepinephrine and triglycerides, and possibly concepts such as glucose toxicity and lipotoxicity, might interact, influence and perhaps explain the perturbed β-cell function seen in this and other studies. Recent data suggests that interesting genes will be discovered in the future; both on Xp and Xq [23], and that expression of these genes will provide new targets for treatment of T2DM, both in TS, but certainly also in a broader T2DM population.

Unexpectedly, and contrary to earlier findings, we found a higher VO_2_max in our patient population [31,4,36]. Still, it seems unlikely that this alone could explain the present differences in glucose metabolism. In addition, the dynamics of high-frequency insulin oscillations were normal as assessed by analysis of minute-to-minute insulin measurements, pointing towards a normal baseline β-cell function. This method has previously been demonstrated to be a sensitive marker of β-cell function [37].

## Conclusions

Thus, the presented data show that neither decreased insulin sensitivity nor significantly decreased β-cell function after stimulation with hyperglycaemia or during challenge with arginine and GLP-1 explain the abnormalities in the glucose homeostasis in TS. However, young women with TS show early discrete signs of decreased β-cell function during testing with OGTT and IVGTT, and the data could be interpreted as a syndrome specific background for the development of diabetes [23], with involvement of multiple variables, such as IGF-I, norepinephrine and triglycerides. We recommend that all women with TS are tested regularly for the presence of diabetes, and we suggest that the high rate of T2DM is due to faltering β-cell function as presented here has a genetic basis.

## Competing interests

The authors declare that they have no competing interests.

## Authors' contributions

All of the authors contributed to the study design, data interpretation, and discussion of study results. BEH and CHG prepared the first draft of the manuscript and subsequently all authors took active part in preparing the final manuscript and were active participants throughout the life of the study. BEH was the principal investigator for the study and together with CHG performed the statistical analysis. CHG, JSC and OS developed the study concept and research questions. CBJ analyzed the insulin pulsatility data. All of the authors read and approve of the final manuscript.

## Pre-publication history

The pre-publication history for this paper can be accessed here:

http://www.biomedcentral.com/1472-6823/11/6/prepub
